# Case Report: New Onset Lymphadenopathy After Immune Checkpoint Inhibitor Therapy Presents a Clinicopathological and Radiological Challenge

**DOI:** 10.3389/fonc.2022.876797

**Published:** 2022-05-20

**Authors:** Matthew Scarlotta, Robin Avery, Ezra Baraban, Zahra Maleki, Yasser Ged

**Affiliations:** ^1^ Department of Oncology, The Johns Hopkins University School of Medicine, Baltimore, MD, United States; ^2^ Department of Medicine, The Johns Hopkins University School of Medicine, Baltimore, MD, United States; ^3^ Department of Pathology, The Johns Hopkins University School of Medicine, Baltimore, MD, United States

**Keywords:** immune checkpoint inhibitors, lymphadenopathy, renal cell carcinoma, diffuse large B-cell lymphoma, sarcoid-like reaction, histoplasmosis, immune-related adverse event

## Abstract

The use of immune-checkpoint inhibitor (ICI) therapy has significantly improved patient outcomes in a wide variety of cancers and has become a cornerstone in the treatment of renal cell carcinoma. However, ICI treatment has the potential to cause a variety of immune-related adverse events (irAEs) that can affect any tissue or organ. This report describes the diagnostic dilemma of a patient with both RCC and diffuse large B-cell lymphoma who developed acute onset of fever and diffuse lymphadenopathy following treatment with combined ipilimumab and nivolumab. While diagnostic considerations included worsening lymphoma, hyperprogression of RCC, sarcoid-like reaction from immunotherapy, and fungal infection, his lymphadenopathy eventually resolved with treatment for histoplasmosis and discontinuation of immunotherapy. Despite only receiving two doses of immunotherapy, he has not required additional systemic therapy for RCC. This case demonstrates both the effectiveness of ICI therapy and the need for multidisciplinary approach to potential irAEs.

## Introduction

Immune-checkpoint inhibitor (ICI) therapy has become integral in the treatment of renal cell carcinoma (RCC) and many other malignancies. Despite the striking survival benefit of these agents, immune-related adverse events (irAEs) can occur in a subset of patients with a wide range of presentations and severity that can be challenging to diagnose and treat. Several management algorithms and guidelines have been developed to guide treatment of irAEs (1, 2). Currently, there are no validated biomarkers to guide treatment selection or stratify patients at risk for developing irAEs. Due to the importance of immediate intervention, providers need to recognize the myriad of ways irAEs can manifest and the diagnostic challenges they pose.

In this report, we present a patient with a history of both RCC and diffuse large B-cell lymphoma (DLCBL) who presents with fever and diffuse FDG-avid lymphadenopathy after treatment with immune checkpoint inhibitors. This case represents a diagnostic challenge due to the broad differential diagnosis including malignant, infectious, and immunotherapy-related causes, and it highlights the importance of a multidisciplinary approach to lymphadenopathy following ICI treatment. The history of both RCC and DLBCL supports a previously identified link between RCC and hematologic malignancies as well as the need to further evaluate the genetic and environmental factors associated with their co-occurrence.

## Case Presentation

A 65-year-old man was initially diagnosed with clear cell renal cell carcinoma (ccRCC) in 2002 after presenting with gross hematuria and underwent left radical nephrectomy showing a 5.5-cm nucleolar grade 2 ccRCC with renal vein invasion, staged as pT3B according to the AJCC sixth edition staging criteria. He was later diagnosed in 2019 with stage II DLBCL, germinal center type with right inguinal and right femoral involvement. During his lymphoma evaluation, he was found to have an incidental 3.2-cm mass in the medial aspect of the right mid kidney and a 1.1-cm mass in the interpolar right kidney suspicious for RCC. Core needle biopsy confirmed recurrent ccRCC.

He received three cycles of R-CHOP chemotherapy and consolidative radiotherapy (RT) for his DLBCL with a posttreatment positron emission tomography/computed tomography (PET/CT) showing complete resolution of previously noted lymphadenopathy. He underwent right partial nephrectomy of the right mid kidney mass and cryoablation of the interpolar right kidney mass. Subsequent imaging showed concern for metastatic disease with a PET/CT scan in June 2020 revealing bilateral pulmonary nodules as well as lesions in the right hepatic lobe, adjacent to the gallbladder, and an L3 lytic lesion. Ultrasound-guided liver biopsy confirmed metastatic ccRCC.

He was treated with stereotactic body radiation therapy (SBRT) to L3 with 2,400 cGy given he was symptomatic with pain at the site of his L3 metastasis. He was subsequently referred to Medical Oncology at the Sidney Kimmel Cancer Center at Johns Hopkins. His disease risk status was intermediate per the International Metastatic Renal Cell Carcinoma Database Consortium (IMDC) risk groups. In this context, he initiated treatment for metastatic ccRCC with doublet immune-checkpoint inhibitors (ICIs) using the combination of ipilimumab 1 mg/kg and nivolumab 3 mg/kg, which is an FDA-approved regimen based on the phase 3 Checkmate-214 clinical trial (3).

**Figure 1:**
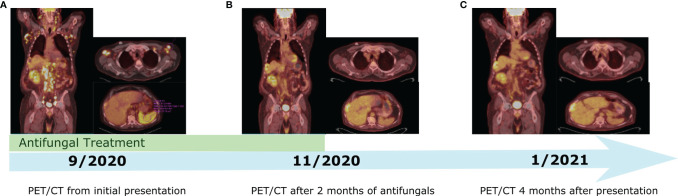
Timeline of PET/CT imaging throughout the patient's course. **(A)** PET/CT from Initial presentation with fever and diffuse FDG avid lymphadenopathy. **(B)** PET/CT showing significant decrease in size and metabolic activity of previously enlarged lymph nodes after two months of antifungal treatment. **(C)** PET/CT showing complete resolution of lymphadenopathy 4 months after initial presentation.

**Figure 2:**
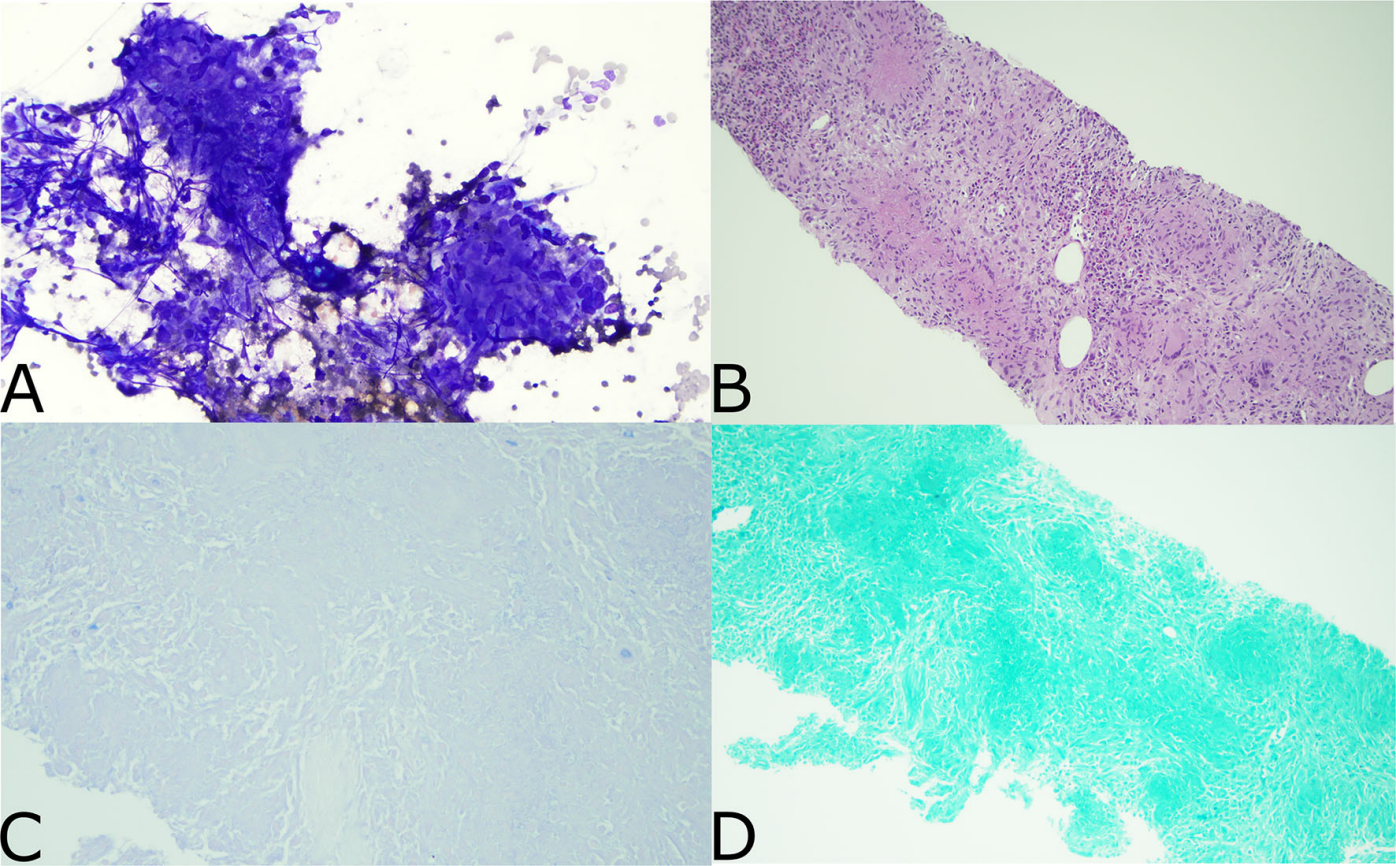
Pathology from Left axillary FNA. **(A)** Aspirated material of a lymph node shows multiple granulomas. They are charecterized by syncytium cytoplasm and spindled, elongated, carrot shaped and barefoot shaped nuclei (Diff-Quik stain, x200). **(B)** This small core biopsy shows numerous necrotizing granulomatous inflammation effacing the entire lymphoid tissue. The granulomas are characterized by round to oval structures consisting of epithelioid histiocyties with focal central necrosis. Scattered lymphocytes and multinucleated giant cell are noted (H&E stain X100). **(C)** Ziehl neelsen stain is negative for mycobacterial organisms (Ziel neelsen stain X400). **(D)** GMS stain is negative for fungal microorganisms (GMS stain X200).

Following his second dose, he presented to the emergency department with non-neutropenic fevers and rigors without a clear source. He continued to have intermittent fevers despite regular acetaminophen given to treat a potential reaction to ipilimumab plus nivolumab. He was admitted to the hospital after returning to the emergency department with persistent fevers, acute kidney injury, and transaminitis. He remained febrile despite broad-spectrum antibiotics and isavuconazole given in the context of his pulmonary nodules. CT imaging showed new generalized progressive lymphadenopathy in the chest, abdomen, and pelvis. PET/CT scan showed extensive, intensely FDG avid lymphadenopathy as well as new diffusely avid splenomegaly (**Figure 1**). In view of this, the differential diagnosis included recurrence of DLBCL, hyperprogression of metastatic ccRCC, immune-related toxicity with sarcoid-like reaction, or other causes of inflammatory and infectious lymphadenopathy. Left axillary fine needle aspirate (FNA) and core biopsy showed granulomatous inflammation with negative stains for mycobacteria (Ziehl–Neelsen stain) or fungal organisms (GMS stain) and were also negative for involvement with RCC, lymphoma, or other malignancy (**Figure 2**). An extensive infectious disease evaluation was negative including T-spot, acid-fast bacillus (AFB) blood culture, coccidioides serologies, Q fever serologies, Bartonella serologies, blood histoplasma antigen, and histoplasma antibody. Given the possibility of histoplasmosis as the cause of his granulomatous inflammation in the context of farm exposures and splenic granulomata, he was transitioned from isavuconazole to posaconazole for a prolonged course despite negative blood histoplasma antigen and antibodies. While a sarcoid-like reaction secondary to ICIs was also considered, steroids were not initiated given normal pulmonary function tests (PFTs) and lack of parenchymal manifestations. His fevers resolved after 1 week of antifungal therapy. He was continued on posaconazole for 3 weeks and transitioned to itraconazole due to lower-extremity edema. Itraconazole was continued until a follow-up PET/CT 2 months after presentation showed a marked decrease in the size and metabolic activity of his previously enlarged cervical, thoracic, abdominal, and pelvic lymph nodes (**Figure 1**). Repeat PET/CT 4 months after presentation showed complete resolution of his lymphadenopathy, which was ultimately attributed to disseminated histoplasmosis (**Figure 1**). Treatment with ICI was discontinued given the small possibility that his presentation could be immune-related. Given he had stable disease and there was concern for ongoing infection, he was monitored off therapy. His RCC remained stable without treatment besides a growing right chest wall metastatic lesion for which he received additional radiotherapy in February 2021. He has remained off systemic therapy without fevers, and his most recent surveillance CT imaging showed stable disease in August 2021 consistent with a treatment-free survival of approximately 12 months since his last dose of ICI. Considering his personal history of two malignancies, he underwent germline genetic testing which was negative for pathogenic alterations.

## Discussion

As in this patient with both RCC and DLBCL, multiple studies have shown an association between RCC and hematologic malignancies (4–10). Concurrent RCC and hematologic malignancies occur mostly in men, typically involving lymphoid malignancies, and the hematologic malignancy usually occurs concurrently or prior to RCC (8, 10). Prior epidemiologic studies have shown a higher than expected occurrence of RCC and non-Hodgkin lymphoma (NHL) with observed-to-expected rates of RCC in the NHL population of 1.86 and observed-to-expected rates of NHL in the RCC population of 2.67 (9). Another SEER analysis observed a significantly higher occurrence of NHL and RCC than expected (4). In this study, the number of RCC cases after an NHL diagnosis was significantly higher than expected with an observed-to-expected ratio of 1.51 (95% CI 1.36–1.66), but the observed number of cases of NHL after RCC was not significantly different.

The mechanism of this association is speculative, but it may be due to common genetic mutations, environmental factors, or immune dysregulation (5–7, 10, 11). The study of relatives of patients with concurrent RCC and hematologic malignancy has suggested a hereditary association given greater lymphoid-predominant malignancy in men within these families and a suggestion of age-of-onset anticipation (12). However, specific mutations linking these concurrent malignancies have not been found thus far. While hereditary renal cancer syndromes such as those involving the Von Hippel–Lindau tumor-suppressor gene have been identified, these syndromes do not typically lead to an increased risk of hematologic malignancies (13). However, there are abnormalities of 3p chromosome that have been reported in B-cell lymphomas (14). This is relevant considering that the Von Hippel–Lindau (VHL) gene is located on the 3p chromosome, and loss of 3p is considered a landmark driver in disease evolution (15, 16). Furthermore, patients with Cowden syndrome characterized by the presence of PTEN germline alterations are at risk for the development of RCC and lymphomas, but this pathway has not been shown to be a definitive common pathway between the two malignancies (11, 17). Others have suggested that dysregulation in immune surveillance in the context of lymphoma may predispose individuals to RCC (4). Further studies are needed to more thoroughly examine the association between RCC and lymphoid malignancies.

Our patient presented with diffuse lymphadenopathy after 2 cycles of ICI combination therapy. In recent years, several small studies have reported the association between ICI and a sarcoid-like reaction often presenting as bilateral mediastinal or hilar lymphadenopathy (18–23). While it has rarely been reported with PD-1/PD-L1 inhibitors, ipilimumab (a monoclonal antibody targeting CTLA-4) is most commonly implicated with around 5% of patients showing suggestive radiologic findings of sarcoidosis after treatment with ipilimumab (18). Patients may also have parenchymal findings but rarely have concomitant abdominal lymphadenopathy. Organ involvement is typically avid on fluorodeoxyglucose PET imaging and therefore difficult to distinguish from malignancy. Our patient also presented with splenic involvement, which can occur in a sarcoid-like reaction since granulomatous infiltration of the spleen can present as homogenous uptake on FDG/PET or small intrasplenic lesions (19, 20). Sarcoid-like reactions typically occur 3–6 months after treatment initiation, and it is often asymptomatic although there may be accompanying dyspnea, cough, skin manifestations, or other systemic symptoms (18, 21, 22). Given the difficulty in differentiating sarcoid-like reaction from malignancy or infectious causes based on imaging, biopsy is often performed. Similarly to sarcoidosis, a biopsy typically reveals non-caseating epithelioid and giant cell granulomas as seen in our patient. In a review of 18 reported cases, ICI was halted in 14 patients and 9 were treated with prednisone. Resolution occurred in all patients except for two who remained stable without treatment (23). While it usually has a benign course, clinicians should be aware of the possibility of sarcoid-like reactions following immunotherapy to prevent misinterpretation of imaging studies as treatment failure or progression.

While a sarcoid-like reaction was considered, the diffuse abdominal lymphadenopathy, persistent fevers, splenic calcified granulomata, and significant farm exposure raised concerns for disseminated histoplasmosis. Previous CT imaging from over 10 years prior showed evidence of splenic granulomata, indicating the possibility for reactivation. Despite the negative blood histoplasma antigen testing, there was a high enough index of suspicion to empirically treat given the limitations of this assay. Notably, a multicenter trial of the enzyme immunoassay-based test for the histoplasma antigen found a sensitivity of 91.8% for disseminated histoplasmosis, but the sensitivity was much lower (30%) for subacute histoplasmosis (24). Testing for the antigen from blood and urine can increase sensitivity (25). His histoplasma antibody was negative as well although his antibody response may have been inhibited secondary to his recent lymphoma and lymphoma treatment. Serious infections are not a common irAE during treatment with ipilimumab and nivolumab. While one study reported a 7.3% incidence of serious infection in patients with melanoma treated with immunotherapy, the main risk factor found in this study was treatment with corticosteroids or infliximab. The risk of serious infection was only 2% in those treated with immunotherapy without corticosteroids or infliximab and mainly consisted of bacterial infections (26). In fact, there is evidence that the PD-1/PD-L1 pathway is critical to fungal immune evasion, and PD-1 inhibition was shown to drastically improve the host immune response in mouse models of histoplasmosis (27).

In this case, the patient was treated empirically for disseminated histoplasmosis given his exposure history and persistent fevers with improvement in his lymphadenopathy on PET scan 2 months after initiating treatment. Given concern for an irAE, ipilimumab and nivolumab were discontinued, but the patient never required steroids given lack of pulmonary symptoms and normal spirometry. Fortunately, even those patients requiring discontinuation of immunotherapy seem to have improved outcomes compared with first-line tyrosine kinase inhibitors. The CheckMate 214 study compared previously untreated patients with RCC randomized to nivolumab and ipilimumab for 4 cycles followed by nivolumab versus treatment with oral sunitinib (3). Treatment-free survival (TFS) was compared between patients who discontinued treatment, and the median TFS was found to favor immunotherapy (28). Our patient did not require additional treatment until progression of a chest wall mass requiring radiation therapy almost 6 months after discontinuation of immunotherapy, and he has not required further systemic therapy.

## Conclusion

With the expanding use of ICIs across multiple solid tumors, many questions remain regarding their optimal use, toxicity management, duration of treatment, and personalized biomarkers, among others. Our case sheds light on the challenges physicians encounter managing new onset lymphadenopathy on ICI therapy considering the wide range of potential etiologies, and it highlights the importance of biopsy of indeterminate lesions as well as the multidisciplinary management of suspected ICI toxicity. In addition, our case illustrates the limitations of diagnostic testing for invasive fungal infections and the importance of clinical suspicion based on environmental exposures and characteristic radiologic findings.

## Data Availability Statement

The original contributions presented in the study are included in the article/supplementary material. Further inquiries can be directed to the corresponding author.

## Ethics Statement

Written informed consent was obtained from the individual(s) for the publication of any potentially identifiable images or data included in this article.

## Author Contributions

MS, YG, and RA were responsible for the concept of the paper and wrote the manuscript. YG and RA treated the patient. ZM and EB interpreted the pathology and provided pathology images for the manuscript. All authors contributed to the article and approved the submitted version.

## Conflict of Interest

The authors declare that the research was conducted in the absence of any commercial or financial relationships that could be construed as a potential conflict of interest.

## Publisher’s Note

All claims expressed in this article are solely those of the authors and do not necessarily represent those of their affiliated organizations, or those of the publisher, the editors and the reviewers. Any product that may be evaluated in this article, or claim that may be made by its manufacturer, is not guaranteed or endorsed by the publisher.
